# The Effect of Mindfulness Training on Proactive and Reactive Cognitive Control

**DOI:** 10.3389/fpsyg.2018.01002

**Published:** 2018-06-20

**Authors:** Yunyun Li, Fang Liu, Qin Zhang, Xinghua Liu, Ping Wei

**Affiliations:** ^1^Beijing Key Laboratory of Learning and Cognition, Department of Psychology, Capital Normal University, Beijing, China; ^2^Academy of Psychology and Behavior, Tianjin Normal University, Tianjin, China

**Keywords:** mindfulness training, proactive control, reactive control, attention skill, FFMQ

## Abstract

Previous studies have demonstrated that mindfulness practice can improve general cognitive control. However, little research has examined whether mindfulness practices affect different cognitive control strategies. According to the dual mechanisms of control (DMC) model, different cognitive control strategies may play distinct roles in individuals’ lives. Proactive control allows people to maintain and prepare for goals, whereas reactive control allows them to respond flexibly to a changing environment. Thus, this study investigates the influences of mindfulness training on proactive and reactive control measured by the AX version of the Continuous Performance Test (AX-CPT). Thirty participants completed AX-CPT and the Five Facet Mindfulness Questionnaire (FFMQ) before and after random assignment to either an 8-week mindfulness training group or a control group. The results showed no interaction between group and test time for AY or BX trial type, but the training group had fewer post-test errors on the BX trial and a higher Behavior Shift Index (BSI) of reaction time (RT) compared with the control group. This finding indicates enhanced trend of proactive control with mindfulness training. A positive correlation between the BSI of RT and observing scores on the FFMQ confirmed the connection between attentional components in mindfulness and proactive control. Errors on the AY trial in the post-test decreased in both groups, reflecting reactive control that did not differ between groups. The 8-week mindfulness training demonstrates a potential improvement effect on proactive control and could be helpful in overcoming interference.

## Introduction

Mindfulness that requires concentration on a current target and adopts open and non-judgmental attitudes toward present-moment experiences has become an extremely influential practice in recent years (Kabat-Zinn, 2003; Van Dam et al., 2018). It is also a major area of research across subdisciplines of psychology (Van Dam et al., 2018). Previous studies have demonstrated that individuals who had mindfulness practice-relevant experiences performed better on some cognitive control tasks such as the Stroop test or similar activities (Wenk-Sormaz, 2005; Chan and Woollacott, 2007; Moore and Malinowski, 2009; Van den Hurk et al., 2010; Allen et al., 2012; Moore et al., 2012; Fan et al., 2015; Fabio and Towey, 2018). These kinds of tasks ask participants to attend to task-relevant information and suppress irrelevant processing or overcome a conflict caused by interference (Miller and Cohen, 2001), reflecting general cognitive control demands.

**FIGURE 1:**
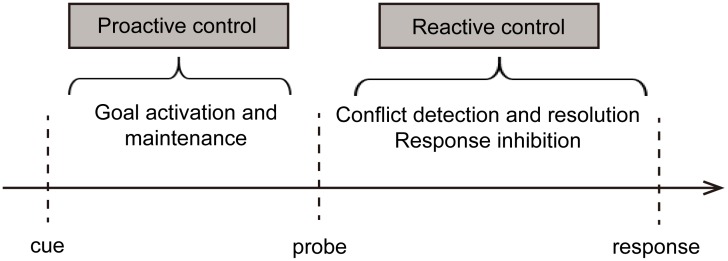
Schematic overview of the cognitive control process in the dual mechanism of control.

Cognitive control is thought to employ multiple processes for task completion, such as focusing attention, meta-cognitive monitoring, and switching between tasks (Slagter et al., 2011). However, the main processes of cognitive control vary across situations. According to the dual mechanisms of control model (DMC; Braver et al., 2007; Braver, 2012), success on control tasks depends on strategies such as proactive or reactive control. The proactive control mode occurs early and persistently. It activates and maintains goal information during the presentation of a cue until critical goal-related stimuli occur, operating in a top-down manner; for instance, a person keeps his dry cleaning in mind during the day and thus declines a colleague’s invitation to go shopping after work. In contrast, the reactive control mode detects potential conflict at a later stage. It corrects responses by transiently recalling goal information as long as task-relevant or interferential stimuli occur, operating in a bottom-up manner. For example, an individual on the way to the cinema suddenly finds a dry-cleaning ticket in her pocket and then proceeds to the dry-cleaner to retrieve her clothes. These two cognitive control modes are engaged in different time courses (see **Figure 1**). The respective influence of proactive and reactive control strategies will change with individual differences or situations (Braver, 2012).

The top-down nature of proactive control results in it being a more voluntary and effective process while demanding greater cognitive resources (Braver et al., 2007, 2009). Therefore, proactive control is vulnerable to cognitive decline such as that accompanying aging (Braver et al., 2005, 2009; Paxton et al., 2006, 2008), schizophrenia (Edwards et al., 2010), and Alzheimer’s disease (Braver et al., 2005). Deficiency in proactive control can impair individuals’ social lives; they may fail to maintain goal progress and prepare for responses. Similarly, a lack of necessary reactive control also poses life-related limitations. For instance, perseveration on top-down goals, which reflects a shortage of reactive control, can lead to habitual bias and hinder the learning of new rules (Munakata et al., 2012). Therefore, developing strong proactive and reactive control abilities is essential to implementing plans and adapting to changing environments. Effective training programs, such as mindfulness practices, can also benefit individuals who have a deficit in proactive or reactive control.

To the best of the authors’ knowledge, few studies have investigated the effects of mindfulness on different cognitive control strategies. One such work demonstrated the facilitation of mindfulness on predictive control in the predictive and reactive control systems (PARCS) (Tops et al., 2014). However, predictive control is different from the proactive or reactive control in which the present study is interested. Proactive control in DMC frameworks (Braver, 2012) is part of reactive control proposed in PARCS. Control by the reactive system in PARCS also includes short-term anticipation (Tops et al., 2014); work by Tops et al. (2014) thus offered no conclusions regarding the potential role of mindfulness in proactive and reactive control in DMC frameworks. Chang et al. (2017) demonstrated that relative to individuals with less mindfulness (Study 1) and those in the control groups (Study 2), individuals with more mindfulness (Study 1) and those in the 10-min mindfulness intervention group (Study 2) showed better and more flexible recruitment of proactive and reactive control. The authors attributed this pattern to the top-down and bottom-up processes involved in mindfulness (Chiesa et al., 2013).

In the authors’ opinion, the effects of mindfulness practices on cognitive control could be related to attention training. Many practice protocols based on mindfulness, such as mindfulness-based stress reduction (MBSR; Kabat-Zinn et al., 1992; Kabat-Zinn, 2003), mindfulness-based cognitive therapy (MBCT; Segal et al., 2002), and integrative body-mind training (IBMT; Tang et al., 2009), stress the core role of attention in mindfulness. Researchers have claimed that two forms of attention are developed during mindfulness practices: concentrative attention and receptive attention (Brown, 1977; Speeth, 1982; Valentine and Sweet, 1999). Concentrative attention is required to direct attention to a focal object, such as one’s breath, whereas receptive attention distributes attention among various stimuli without orientation or guidance. Empirical studies have examined the role of mindfulness on attention tasks. Some studies reported that performance on sustained attention tasks requiring concentrative attention was better in individuals who had experienced mindfulness training than in those without prior mindfulness experiences (Pagnoni and Cekic, 2007; Lutz et al., 2009; Schmertz et al., 2009; MacLean et al., 2010; Galla et al., 2011; Tarrasch et al., 2016). Other work showed that mindfulness promoted attention flexibility, which required receptive attention to detect and handle new stimuli (Heeren et al., 2009; Moore and Malinowski, 2009; Greenberg et al., 2012). Facilitated performance on attention tasks thus illustrated that the abilities of concentrative attention and receptive attention were improved during mindfulness practices.

Theoretically, improved attention skills might contribute to more successful cognitive control because effective and flexible cognitive control depends on the ability to focus continuously on a target and detect important new changes in the environment. Furthermore, specific functions of different cognitive control strategies might be associated with distinct attention processes. The function of proactive control is to maintain goal-related information (Braver et al., 2007; Braver, 2012); it acts in an anticipatory manner and recruits sustained attention on a focused object, hence correlating with concentrative attention. In contrast, the function of reactive control is to temporally reactivate a goal-related context when a crucial event occurs (Braver et al., 2007; Braver, 2012); it acts in a transient manner and recruits flexible attention to detect and shift to new stimuli, therefore associating with receptive attention. In other words, proactive control and reactive control may rely on concentrative attention and receptive attention, respectively. Given that previous studies demonstrated improved concentrative attention and receptive attention during mindfulness practices, we infer that mindfulness practices could be an effective way to enhance proactive control and reactive control. Findings by Chang et al. (2017) concur with this hypothesis. Their observation of better proactive and reactive control associated with mindfulness might be attributed to the improvement of concentrative attention and receptive attention in mindfulness training; however, research by Chang et al. (2017) did not use a standardized mindfulness training method. A systemic mindfulness training is thus necessary to explore the effects of mindfulness on proactive and reactive control strategies.

This study aims to investigate the effects of an 8-week mindfulness training on different cognitive control modes. As concentrative attention and receptive attention are each trained during mindfulness, it is reasonable to assume that mindfulness practices can facilitate different cognitive control modes. We expected that over an 8-week mindfulness practice, training on attention would promote cognitive control of context maintenance and the ability to overcome distractors. The protocol was also expected to enhance one’s flexibility to detect and attend to new stimuli. In other words, we anticipated that proactive and reactive control modes would both be enhanced through mindfulness training.

## Materials and Methods

### Participants and Procedure

Using G^∗^Power 3.1.9.2, the number of participants required for the interaction of test time by group was calculated based on Fan et al. (2015, ω^2^ = 0.17; setting 1-β = 0.95). Result showed that a total of 18 participants were needed to achieve adequate test power.

A total of 34 individuals without mindfulness-related experience participated in this project at a public lecture about mindfulness in the Beijing area. Participants were guided through a mindfulness practice for 5 min under the direction of an instructor who introduced the benefits of mindfulness on mental health and emotion but did not mention the effect on attention. All participants had normal or corrected-to-normal vision. All subjects gave written informed consent in accordance with the Declaration of Helsinki. The protocol was approved by the Institutional Review Board of the Capital Normal University.

To examine the effects of mindfulness training on proactive and reactive control simultaneously, we adopted the AX version of the Continuous Performance Test (AX-CPT; Servan-Schreiber et al., 1996), a well-established paradigm for studying the strength difference between proactive and reactive cognitive control. In the pre-training tests, all participants completed the AX-CPT and filled out the Chinese revised version (Deng et al., 2011) of the Five Facet Mindfulness Questionnaire (FFMQ; Baer et al., 2006), which was used to measure their mindfulness levels. The presentation order of the questionnaire and cognitive tasks was balanced across subjects. Then, participants were randomly assigned to either the 8-week mindfulness training group or the waitlist control group. Eight weeks later, both groups received a post-training test containing the same procedure as the pre-training test. A 2-day intensive mindfulness training was administered to the control group immediately after the post-test. Two participants responded incorrectly on the AY trial type (see section “AX-CPT Task”), and two individuals had not finished all training or the post-test, so the final sample consisted of 15 participants (5 males) in the training group and 15 participants (5 males) in the control group. The difference in mean age between training group (*M* = 30.4, *SD* = 8.8) and control group (*M* = 28.4, *SD* = 9.7) was not significant, *t*(28) = 0.59, *p* = 0.56, BF_01_ = 2.54.

### Five Facet Mindfulness Questionnaire (FFMQ)

The self-report measure of the FFMQ consists of 39 items assessing five factors, classified into the following subscales: Observing, Describing, Acting with awareness, Non-judging, and Non-reacting. Items are scored on a 5-point Likert-type scale (1 = *never or very rarely true*, 5 = *very often or always true*). Some items are reverse-scored. When comparing between groups or between pre- and post-tests, higher scores indicate a more advanced level of mindfulness. Scores on the 39 items and five subscale items showed good internal consistency for 30 valid participants on the pre-test (Cronbach’s α = 0.84, 0.85, 0.83, 0.86, 0.80, and 0.83, respectively) and post-test measures (Cronbach’s α = 0.87, 0.92, 0.85, 0.94, 0.81, and 0.88, respectively).

### AX-CPT Task

**FIGURE 2:**
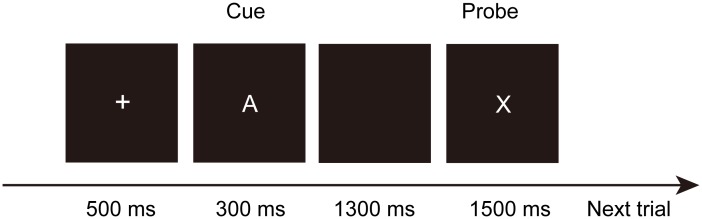
Sample trial procedure in AX-CPT.

Each trial started with a fixation cross for 500 ms, then the cue letter appeared on the screen for 300 ms followed by a delay period of 1300 ms. After that, the probe letter appeared for 1500 ms (see **Figure 2**). Participants were asked to provide a target response to an X probe letter following an A cue (i.e., AX trial). A non-target response was required for the other three non-target trial types: cue A followed by a “non-X” (referred to as Y) probe letter (i.e., AY trial), a “non-A” (referred to as B) cue followed by an X probe letter (i.e., BX trial), and a “non-A” cue followed by a “non-X” probe letter (i.e., BY trial). The left and right buttons of the mouse were set to target and non-target response buttons, which were counterbalanced across participants. Participants were asked to respond to the probe as correctly and rapidly as possible. The entire task included two blocks with a 5-min break between blocks. Each block consisted of 70 AX trials, 10 AY trials, 10 BX trials, and 10 BY trials. In this study, the letters A and X were used as the target cue and probe, respectively. Non-target cues and probes were randomly selected from the letters B, D, E, F, G, M, P, S, U, and Z. Identical cues and probes, such as a trial composed of cue D and probe D, were not allowed in a single trial. Each participant practiced 20 trials prior to the formal test.

In the AX-CPT task, because the proportion of the target trial type (70%) was much higher than that of the other three non-target trial types (10% each), cue A and probe X could induce a strong target response trend. In the non-target trials AY and BX, conflicts caused by the target response trend to cue A and probe X needed to be overcome to ensure correct responses. When proactive control is dominant, individuals are likely to make a target response for cue A and a non-target response for cue B. Thus, the advantage of dominant proactive control is that the enhanced non-target trend for cue B facilitates a consequent response to probe X, resulting in a lower error rate and/or faster RT. The disadvantage of dominant proactive control is that a non-target probe Y following cue A is more likely to induce a poor reaction (i.e., a high error rate and/or slow RT). In contrast, when reactive control is dominant, maintenance of cue information is attenuated. Therefore, individuals were expected to perform well on the AY trial but poorly on the BX trial. As the balance between AY and BX reflects the shift between proactive and reactive control, the Behavior Shift Index (BSI; Braver et al., 2009; Edwards et al., 2010) was used to measure an individual’s control style. The BSI, a combination of AY and BX trials for error rate or RT, was calculated by (AY – BX)/(AY + BX). Positive values of the indices indicate strong use of proactive control, whereas negative scores indicate poor use of proactive control. A higher positive score implies stronger use of proactive control when comparing groups or between pre- and post-test (Braver et al., 2009; Edwards et al., 2010). Trials with a 0 error rate were corrected as (error + 0.5)/(frequency of trials + 1).

### Mindfulness Practice

The mindfulness training was designed based on the protocol for MBCT (Segal et al., 2002), emphasizing a focus on the present and non-judgmental awareness. Depression-related content was not suitable for non-clinical individuals in this study; therefore, relevant sections were replaced by other meditation practices from MBSR, such as mindfulness yoga (Kabat-Zinn, 1982, 1990). The intervention lasted 8 weeks and consisted of a 2.5-h group training each week. Mindfulness practices included (1) body scans (paying attention to the sensation of body from head to toe); (2) sitting meditation (focusing on and experiencing one’s own breath or thought when in a comfortable sitting position); (3) walking meditation (observing and experiencing the sensation of moving parts of the body); and (4) mindfulness yoga (focusing on and maintaining stretching). Together, these forms of mindfulness practices aim to improve one’s ability to observe transitory thoughts and physical sensations in the present moment. No less than 30 min of home exercise was recorded daily by self-report in a notebook. Participants’ feelings and thoughts were shared, and necessary guidance was provided during group discussions. The instructors, who were blind to the purpose of the experiment, had over 4 years of personal experience in mindfulness and more than 2 years of group teaching experience.

### Data Analysis

Inaccurate and missing responses were used to calculate the error rate. Correct trials with RTs shorter than 150 ms or longer than 1100 ms were excluded (1.3% of correct trials). We performed a series of *t*-tests and repeated-measures analyses of variance (ANOVAs) on FFMQ scores, error rates, RTs, and BSIs. Greenhouse-Geisser corrected results were adopted when necessary. Multiple contrasts were corrected using the Bonferroni method. Bayes Factors (BF) were calculated using JASP 0.8.6 statistical package (JASP Team, 2018; Wagenmakers et al., 2018). BF_10_ was reported when statistical test was significant or marginal significant (BF_10_ = the marginal likelihood of the alternative hypothesis/the marginal likelihood of the null hypothesis). BF_01_ was reported when statistical test was insignificant (BF_01_ = the marginal likelihood of the null hypothesis/the marginal likelihood of the alternative hypothesis).

In the exploratory analysis, a 3-way ANOVA revealed no significant 2 (group: training vs. control) × 2 (test time: pre- vs. post-test) × 4 (trial type: AX, AY, BX, BY) interaction for error rate, *F*(3,84) = 0.96, *p* = 0.42, BF_01_ = 3.23, or for RT, *F*(3,84) = 0.14, *p* = 0.93, BF_01_ = 2.71. Planned contrasts (Keppel, 1991) were performed to explore potentially significant results. According to previous research (Paxton et al., 2006), performance on AY and BX trials is the most sensitive indicator of the use of proactive and reactive control. Thus, to simplify the analysis, we examined only AY and BX trial types. A series of 2 (group: training vs. control) × 2 (test time: pre- vs. post-test) ANOVAs were run separately for AY and BX trial performance with an expected interaction of group × test time. We also tested the correlations of FFMQ scores with BSI to investigate the potential connection between mindfulness training and cognitive control. Correlation analysis supplemented the ANOVA results.

## Results

### FFMQ Scores

A 2 (group: training vs. control) × 2 (test time: pre- vs. post-test) repeated-measures ANOVA was conducted on total FFMQ scores (see **Table 1** for total and subscale scores and for *t*-test results of subscale scores), using the group as a between-subjects factor and test time as a within-subjects factor. The main effect of test time was significant, *F*(1,28) = 36.63, *p* < 0.001, $\eta_{p}^{2}$ = 0.57, 1-β = 0.99, BF_10_ = 468.57. The main effect of the group was not significant *F*(1,28) = 0.08, *p* = 0.78, BF_01_ = 2.82. The interaction effect of group by test time was significant, *F*(1,28) = 20.34, *p* < 0.001, $\eta_{p}^{2}$ = 0.42, 1-β = 0.99, BF_10_ = 165.24. Simple effect analysis showed a higher trend for the training group than the control group in post-test scores (*p* = 0.05, *d* = 0.75, BF_10_ = 1.59) and no group difference in pre-test scores (*p* = 0.11, BF_01_ = 1.03). Furthermore, scores were higher on the post-test than on the pre-test for the training group (*p* < 0.001, *d* = 0.99, BF_10_ = 601.59). Results revealed no significant difference between pre- and post-tests for the control group (*p* = 0.09, BF_01_ = 1.03).

**Table 1:** Scores of FFMQ for training and control groups in the pre- and post-test.

	Training group				Control group				Difference between groups (*p*)			
				
FFMQ	Pre-test Mean (*SD*)	Post-test Mean (*SD*)	Difference (*p*)	BF	Pre-test Mean (*SD*)	Post-test Mean (*SD*)	Difference (*p*)	BF	Pre-test	BF	Post-test	BF
Total score	116.1 (13.4)	137.1 (17.6)	21 (<0.001)^∗∗∗^	601.59	123.8 (11.9)	126.9 (7.9)	3.1 (0.09)	1.03	-7.7 (0.11)	1.03	10.2 (0.05)	1.59
Observing	23.1 (4.1)	28.5 (4.9)	5.4 (0.001)^∗∗^	38.83	23.1 (5.3)	22.1 (5.6)	-1 (0.37)	2.64	0 (1)	2.90	6.4 (0.002)^∗∗^	14.98
Describing	25.3 (4.2)	29.6 (5.2)	4.3 (<0.001)^∗∗∗^	84.87	26.9 (5.0)	27.2 (4.2)	0.3 (0.65)	3.46	-1.6 (0.35)	2.07	2.4 (0.18)	1.41
Acting with awareness	25.5 (4.5)	27.9 (6.8)	2.4 (0.17)	1.60	28.8 (5.7)	31.1 (5.4)	2.3 (0.037)^∗^	1.95	-3.3 (0.09)	0.95	-3.2 (0.16)	1.35
Non-judging	23.8 (5.8)	28.1 (5.2)	4.3 (0.002)^∗∗^	23.49	25.5 (4.1)	28.2 (4.5)	2.7 (0.011)^∗^	5.29	-1.7 (0.37)	2.13	-0.1 (0.94)	2.90
Non-reacting	18.4 (3.6)	23.0 (2.9)	4.6 (0.001)^∗∗^	29.21	19.6 (5.4)	18.3 (5.2)	0.7 (0.19)	1.76	-1.2 (0.48)	2.39	4.7 (0.004)^∗∗^	9.72


*SD, standard deviation. ^∗^Indicates a significant difference between groups or within groups according to paired or independent *t*-test comparisons. ^∗^*p* < 0.05, ^∗∗^*p* < 0.01, ^∗∗∗^*P* < 0.001. BF, Bayes Factor. When *p* ≤ 0.05, BF_*10*_ is provided (BF_*10*_ = the marginal likelihood of the alternative hypothesis/the marginal likelihood of the null hypothesis). When *p* > 0.05, BF_*01*_ is provided (BF_*01*_ = the marginal likelihood of the null hypothesis/the marginal likelihood of the alternative hypothesis).*

### Cognitive Task Results

#### Error Rates

The mean error rates and RT of AX-CPT in the two groups at pre- and post-test are listed in **Table 2**. The analysis for error rates on AY trial type showed only a significant main effect of test time, *F*(1,28) = 10.65, *p* = 0.003, $\eta_{p}^{2}$ = 0.28, 1-β = 0.88, BF_10_ = 14.08. Further tests illustrated fewer errors on the AY trial in the post-test than the pre-test for the training group (*p* = 0.026, 1-β = 0.83, BF_10_ = 2.59) and the control group (*p* = 0.027, 1-β = 0.82, BF_10_ = 2.52). There was no significant main effect of group, *F*(1,28) = 0.004, *p* = 0.95, BF_01_ = 2.84, or interactive effect between group and test time, *F*(1,28) = 1.76, *p* = 0.20, BF_01_ = 1.42.

**Table 2:** Proportion of errors (%) and RTs (ms) for training and control groups in pre- and post-test.

	Training group		Control group	
		
	Pre-test Mean (*SE*)	Post-test Mean (*SE*)	Pre-test Mean (*SE*)	Post-test Mean (*SE*)
**Error rate**				

AX	1.2 (0.4)	0.8 (0.2)	2.0 (0.4)	1.1 (0.2)
AY	22.3 (5.5)	7.3 (3.1)	17.7 (5.5)	11.3 (3.1)
BX	13.3 (5.9)	0.7 (1.1)	9.9 (5.9)	2.7 (1.1)
BY	2.4 (1.1)	0.7 (0.6)	3.1 (1.1)	1.7 (0.6)

**RT**				

AX	392 (17)	392 (16)	390 (17)	422 (16)
AY	531 (23)	519 (18)	515 (23)	537 (18)
BX	317 (29)	317 (21)	354 (29)	385 (21)
BY	354 (31)	324 (21)	369 (31)	387 (21)


*SE, standard error.*

The ANOVA for error rates on BX trial type revealed only a significant main effect of test time, *F*(1,28) = 6.62, *p* = 0.016, $\eta_{p}^{2}$ = 0.19, 1-β = 0.71, BF_10_ = 5.42. Further contrasts indicated that, for the training group, errors on the BX trial exhibited a decreasing trend in the post-test relative to the pre-test (*p* = 0.064, 1-β = 0.65, BF_10_ = 1.27). For the control group, the difference between pre- and post-tests was far from statistically significant (*p* = 0.13, BF_01_ = 1.32). There was no significant main effect of group, *F*(1,28) = 0.02, *p* = 0.88, BF_01_ = 3.04, or interactive effect between group and test time, *F*(1,28) = 0.51, *p* = 0.48, BF_01_ = 2.35.

#### RT

The ANOVA results for RTs on AY trial type showed that there was no significant main effect of group, *F*(1,28) = 0.001, *p* = 0.97, BF_01_ = 2.15, or main effect of test time, *F*(1,28) = 0.20, *p* = 0.66, BF_01_ = 3.67, or interactive effect between group and test time, *F*(1,28) = 2.3, *p* = 0.14, BF_01_ = 1.23. For RTs on BX trial type, there was no significant main effect of group *F*(1,28) = 2.68, *p* = 0.11, BF_01_ = 0.95, or main effect of test time, *F*(1,28) = 1, *p* = 0.33, BF_01_ = 2.51, or interactive effect between group and test time, *F*(1,28) = 1.04, *p* = 0.32, BF_01_ = 2.07.

### The BSI

A series of 2 (group: training vs. control) × 2 (test time: pre- vs. post-test) repeated-measures ANOVAs were conducted for the BSIs of the error rate and RT (see **Table 3**). Only a marginally significant group main effect was observed for the BSI of RT, *F*(1,28) = 4.0, *p* = 0.055, $\eta_{p}^{2}$ = 0.13, 1-β = 0.42, BF_10_ = 2.43, revealing a higher trend of BSI for the training group than for the control group. Planned comparisons revealed that the training group had a higher BSI score than the control group on the post-test, *t*(28) = 2.49, *p* = 0.019, *d* = 0.91, 1-β = 0.87, BF_10_ = 3.18. No significant difference appeared between the two groups on the pre-test, *t*(28) = 1.33, *p* = 0.19, BF_01_ = 1.5. For the BSI of RT, there was no significant main effect of test time, *F*(1,28) = 1.89, *p* = 0.18, BF_01_ = 1.78, or the interactive effect between group and test time, *F*(1,28) = 0.30, *p* = 0.59, BF_01_ = 2.53. For the BSI of error rate, there was no significant main effect of group, *F*(1,28) = 0.004, *p* = 0.95, BF_01_ = 3.01, or main effect of test time, *F*(1,28) = 0.19, *p* = 0.67, BF_01_ = 3.46, or interactive effect between group and test time, *F*(1,28) = 0.21, *p* = 0.65, BF_01_ = 2.66.

**Table 3:** Behavioral Shift Index (BSI) for training and control groups in the pre- and post-test.

	Training group			Control group			Difference between groups (*p*)	
			
	Pre-test Mean (*SE*)	Post-test Mean (*SE*)	Difference (*p*)	Pre-test Mean (*SE*)	Post-test Mean (*SE*)	Difference (*p*)	Pre-test	Post-test
Error rate	0.22 (0.10)	0.31 (0.10)	0.09 (0.61)	0.27 (0.10)	0.27 (0.10)	0 (0.98)	-0.05 (0.77)	0.04 (0.77)
RT	0.26 (0.03)	0.25 (0.02)	0.01 (0.43)	0.20 (0.02)	0.17 (0.02)	-0.03 (0.28)	0.06 (0.19)	0.08 (0.019)^∗^


*^∗^Indicates a significant difference between groups or within groups according to paired or independent *t*-test comparisons. Positive values on the BSI indicate strong use of proactive control, whereas negative scores indicate poor use of proactive control. ^∗^*p* < 0.05.*

### Correlation Between FFMQ Scores and BSI

As showed in **Table 4**, correlations were not significant for mindfulness scores and BSI on the pre-test (*p*s > 0.05). The analysis in the post-test showed that the BSI of error rate was negatively associated with the scores of Acting with awareness subscale (*r* = -0.46, *p* = 0.01, BF_10_ = 5.47), and with the scores of Non-judging subscale (*r* = -0.36, *p* = 0.049, BF_10_ = 1.44). The BSI of RT was positively associated with the scores of the observing subscale (*r* = 0.38, *p* = 0.037, BF_10_ = 1.81). The other correlations in the post-test were insignificant (*p*s > 0.05).

**Table 4:** Correlation scores (*r*), and *p*-value between FFMQ scores and Behavioral Shift Index (BSI) in the pre- and post-test.

	Pre-test						Post-test					
		
	BSI of error rate			BSI of RT			BSI of error rate			BSI of RT		
				
FFMQ	*r*	*p*	BF	*r*	*p*	BF	*r*	*p*	BF	*r*	*p*	BF
Total score	-0.2	0.29	2.60	0.07	0.72	4.15	-0.34	0.07	0.91	0.17	0.38	3.03
Observing	0.04	0.85	4.33	0.15	0.44	3.32	0.15	0.42	3.23	0.38	0.037^∗^	1.81
Describing	-0.33	0.07	0.94	0.17	0.38	3.07	0.01	0.98	4.41	0.13	0.50	3.56
Acting with awareness	-0.2	0.28	2.52	-0.07	0.70	4.11	-0.46	0.01^∗^	5.47	-0.14	0.46	3.40
Non-judging	-0.09	0.64	3.96	-0.09	0.63	3.93	-0.36	0.049^∗^	1.44	0.19	0.31	2.68
Non-reacting	0.07	0.72	4.14	0.06	0.74	4.18	-0.25	0.19	1.92	0.26	0.16	1.72


*^∗^Indicates a significant correlation. ^∗^*p* < 0.05. BF, Bayes Factor. When *p* ≤ 0.05, BF_*10*_ is provided (BF_*10*_ = the marginal likelihood of the alternative hypothesis/the marginal likelihood of the null hypothesis). When *p* > 0.05, BF_*01*_ is provided (BF_*01*_ = the marginal likelihood of the null hypothesis/the marginal likelihood of the alternative hypothesis).*

## Discussion

The present study investigated whether an 8-week mindfulness practice modulate proactive or reactive control modes. FFMQ scores showed the 8-week practice led to significant improvements in the mindfulness levels of the training group. These results are in line with those of a previous study by Xu et al. (2016), who found improved mindfulness levels after a 6-week mindfulness training using the same procedure as in this study. Additionally, the results revealed a negative correlation between BSI of error rate and Acting with awareness scores, and with Non-judging subscale scores, whereas a positive correlation between BSI of RT and Observing scores in the post-test, coincident with previous research suggesting that components of mindfulness have dissociable cognitive correlates (Anicha et al., 2011).

The present study adopted the AX-CPT task to measure changes in two forms of cognitive control. The results revealed only a reduced trend of errors on BX trials after training for the mindfulness group, reflecting a pattern of proactive control enhancement. When combining AY and BX trials, the planned comparison for BSI of RT showed a larger positive value for the mindfulness training group relative to the control group at post-test but not at pre-test. As BSI reflects a relative balance between proactive control and reactive control (Braver et al., 2009), this difference between groups implies a stronger proactive control dominance for the mindfulness training group compared to the control group after training. However, because no significant interaction between group and test time was observed, we can only state that an 8-week mindfulness practice has the potential to improve proactive control in the present study. What’s more, reactive control was improved for the mindfulness training group and control group, reflected by significantly reduced errors on AY trial type in the post-test compared to in the pre-test, but no difference between groups was found. A plausible explanation is that reactive control enhancement in both groups might have resulted from a practice effect on the tests.

Compared to findings by Chang et al. (2017) revealing that mindfulness facilitated flexibility in recruiting proactive and reactive control, we only observed potential improvement effects of 8-week mindfulness training on the proactive control mode. The enhanced trend of proactive control in the mindfulness group could be associated with the concentrative attention developed during mindfulness practices. This assumption was partly supported by the positive correlation between BSI of RT and the Observing subscale scores in the post-test. As Observing and Non-reacting subscales are the most widely agreed upon components of mindfulness in previous works (Anicha et al., 2011; Noone et al., 2016), Observing scores were considered representative of the ‘awareness of the present situation,’ while Non-reacting scores were thought representative of ‘the non-reactive monitoring of experiences’ component of mindfulness (Noone et al., 2016). The positive correlation between BSI of RT and the Observing subscale scores reflected a connection between attention in mindfulness and proactive control. It should be noted that, as Acting with awareness and Non-judging subscales are not the most components of mindfulness (Anicha et al., 2011; Noone et al., 2016), there is no further discussion on the correlations between these two subscales and BSI here.

One might argue that this proactivity trend is due to the mindfulness group having received 8 weeks of training whereas the control group simply waited during the period before the post-test. Interest in the group training itself, rather than the training method and content, led training group participants to recruit more attention than the waitlist control participants on the cognitive control task on the post-test (MacLean et al., 2010; MacCoon et al., 2014). Excellent cognitive control depends more on attention skills (Slagter et al., 2011) than on the amount of attention. For example, proactive inhibition control performance on the stop signal task was improved from baseline to post-training in a task-relevant training group but did not differ in an active sham-training control group that received 2-alternative forced-choice RT task training (Berkman et al., 2014). Thus, more attention engagement is likely not responsible for proactive control improvement.

Reactive control was improved in both groups, but no difference between groups was found; we could not conclude that 8-week mindfulness training enhanced reactive control. However, we also could not claim that mindfulness training exerted no effect on reactive control. One possible interpretation is that 8 weeks of mindfulness training may have the potential to promote proactive control, which acts in a top-down manner, whereas a longer duration of mindfulness practice would increase reactive control, which acts in a bottom-up manner. As mentioned by Chiesa et al. (2013), mindfulness practitioners with relatively brief practice experience showed more top-down processes, and those with long-term practice experience showed more bottom-up processes. Furthermore, proactive control is supposed to be more strongly correlated with concentrative attention, whereas reactive control is associated with receptive attention. Previous articles assumed that the development of different attention during mindfulness practices was not synchronized. For example, mindfulness novices have been shown to practice concentrative attention at the beginning of mindfulness training and then develop receptive attention after acquiring concentrative attention at a high level of mindfulness (see Lutz et al., 2007, for a review). That is, mindfulness attention is oriented to and remains around selected objects early in training. With regard to a higher stage of practice, attention can be flexibly disengaged from focus through monitoring anything that occurs in the present without reacting (Lutz et al., 2008). Therefore, the absence of reactive control enhancement in the 8-week mindfulness training group compared with the control group was speculatively associated with the training duration. Although several previous studies showed facilitation of reactive control or flexibility in mindfulness groups, the variety of procedures and tasks must be considered. For instance, Chang et al. (2017) noted both proactive and reactive control modes were improved for participants in a 10-min mindfulness practice group, but this result might relate to the participants’ state of mindfulness immediately after the interventions. Several studies also reported that attention flexibility on which reactive control relied was facilitated in mindfulness groups (Heeren et al., 2009; Moore and Malinowski, 2009; Greenberg et al., 2012). However, given that cognitive tasks differed across these studies (including our study), caution is required when comparing research conclusions. As such, the effects of mindfulness training longer than 8 weeks on reactive control require further investigation.

Results of the present study should be viewed carefully due to the small sample size. A sufficient sample size is needed in subsequent studies to investigate the long-term effects of mindfulness practice on cognitive control. Another limitation is that participants’ expectancy in the training group might have a relationship with proactive or reactive control. Future studies could explore and rule out expectancy effects on cognitive control performance. With the current intervention, we also cannot exclude the possibility that other factors apart from mindfulness, such as physical activity, were responsible for the potential improvement in proactive control. For instance, the effects of yoga on cognition have been widely examined in recent years (see Gothe and McAuley, 2015, for a review). Therefore, future studies should examine the respective effects of various factors on mindfulness training programs with regard to different cognitive control modes.

Considering the potential promotion of mindfulness training on proactive control in this study, it is valuable to popularize and apply this method. Mindfulness training includes the use of daily activities for practice and oral guidance, making it suitable for various populations. Thus, mindfulness practices have the potential to benefit individuals who need to improve proactive control, such as those who are aging (Paxton et al., 2006; Braver et al., 2009) or suffer from schizophrenia (Edwards et al., 2010). The beneficial effects of mindfulness on people with impaired proactive control warrant continued investigation.

## Conclusion

The present study examined the influences of mindfulness on proactive and reactive control modes using the AX-CPT. Proactive control showed a trend of improvement after an 8-week mindfulness practice for the training group; however, enhanced reactive control for the training and control groups could not be attributed directly to the 8-week mindfulness practice. We speculated that the effects of mindfulness on these two different control modes were likely modulated by the duration of mindfulness training. Moreover, awareness of the present in mindfulness, as reflected by scores on the Observing subscale of the FFMQ, were positively correlated with the proactive control mode, providing further evidence of a relationship between components of mindfulness and different aspects of cognition. Consequently, an 8-week mindfulness practice may be helpful in overcoming interference and effectively navigating daily life.

## Author Contributions

QZ, XL, and PW contributed to the conception and design of the study. FL collected the data. YL performed the statistical analysis and wrote the first draft of the manuscript. All authors contributed to manuscript revision, read and approved the submitted version.

## Conflict of Interest Statement

The authors declare that the research was conducted in the absence of any commercial or financial relationships that could be construed as a potential conflict of interest.
